# Fast Light-Driven Motion of Polydopamine Nanomembranes

**DOI:** 10.1021/acs.nanolett.1c03165

**Published:** 2021-12-14

**Authors:** Thomas Vasileiadis, Tommaso Marchesi D’Alvise, Clara-Magdalena Saak, Mikolaj Pochylski, Sean Harvey, Christopher V. Synatschke, Jacek Gapinski, George Fytas, Ellen H. G. Backus, Tanja Weil, Bartlomiej Graczykowski

**Affiliations:** † Faculty of Physics, Adam Mickiewicz University, Uniwersytetu Poznanskiego 2, 61-614 Poznan, Poland; ‡ Max Planck Institute for Polymer Research, Ackermannweg 10, 55128 Mainz, Germany; § Department of Physical Chemistry, University of Vienna, Währinger Strasse 42, 1090 Vienna, Austria

**Keywords:** Photoactuation, soft actuators, artificial muscles, bioinspired materials, nanomembranes, polydopamine

## Abstract

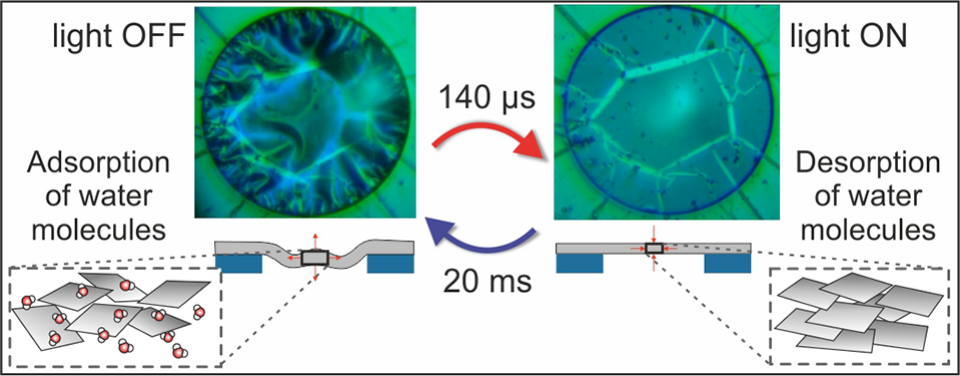

The actuation of micro- and nanostructures controlled by external stimuli remains one of the exciting challenges in nanotechnology due to the wealth of fundamental questions and potential applications in energy harvesting, robotics, sensing, biomedicine, and tunable metamaterials. Photoactuation utilizes the conversion of light into motion through reversible chemical and physical processes and enables remote and spatiotemporal control of the actuation. Here, we report a fast light-to-motion conversion in few-nanometer thick bare polydopamine (PDA) membranes stimulated by visible light. Light-induced heating of PDA leads to desorption of water molecules and contraction of membranes in less than 140 μs. Switching off the light leads to a spontaneous expansion in less than 20 ms due to heat dissipation and water adsorption. Our findings demonstrate that pristine PDA membranes are multiresponsive materials that can be harnessed as robust building blocks for soft, micro-, and nanoscale actuators stimulated by light, temperature, and moisture level.

## Introduction

A skeletal muscle tissue, which has been optimized over hundreds of millions of years of evolution, represents the role model for the design and synthesis of soft materials serving as artificial muscles and actuators. Notably, even the most complex motion results from simple contractions of antagonistic muscle pairs transferred to the skeleton via tendons.^1^ From a mechanical point of view, muscle fiber contraction is more favorable as the expansion can lead to instability and fiber buckling.^2^ The actuators efficiency and versatility require discernible displacement at low energy input^3^ and multiresponsive behavior,^4^ respectively. Other crucial factors include their operational temperature that needs to be minimized to avoid degradation, as well as their response time and weight-lifting ability.^5^ The external stimuli for actuation include heat, light, electric fields and currents, changes in humidity, and exposure to vapors. Light is advantageous over other stimuli for remote and spatiotemporal control of the actuation.

The search for photoresponsive materials for artificial muscles mimicking their natural counterparts faces several trade-offs between light-to-motion conversion efficiency, light wavelength, dynamics, biocompatibility, operational temperature and environment, flexibility, multifunctionality, simplicity, and cost, to name a few.^6−9^ To date, the vast majority of photoactuators employed photochemical and photothermal effects.^10^ In photochemical materials, light-activated molecular level transformations (e.g., cis–trans isomerization, ring-opening and ring-closing, bond exchange, cycloadditions) can lead to macroscopic changes of dimensions or shape. Light-induced heating in photothermal materials can result in positive or negative thermal expansion, phase transitions, or adsorption/desorption of molecules, which are converted into mechanical motion. Typically, photothermal actuators merge light-absorbing heaters (e.g., dyes and semiconducting or plasmonic nanostructures) with thermoresponsive materials such as liquid crystal elastomers, hydrogels, shape memory polymers, and inorganic compounds with volume-changing structural phase transitions.^9,11−14^

In this work, we explore potential photochemical and photothermal effects in polydopamine (PDA). PDA is a multifunctional, bioinspired polymer with diverse applications for biomedical^15,16^ and environmental^17^ purposes, catalysis^18,19^ and photocatalysis,^20,21^ sensing,^22^ photonics^23^ and optoelectronics.^24^ Notably, PDA has excellent photothermal properties over the entire visible spectrum,^25^ similar as the closely related analogues of the melanine family,^26,27^ and remains structurally stable up to 400 K.^28^ These features were utilized in composite photoactuators combining PDA heaters (nanoparticles, thin films) with thermoresponsive polymers.^29−31^ Furthermore, the hydrophilic^28^ and water-swelling properties of PDA-reduced graphene oxide (PDA-RGO) composites were harnessed in bilayers or thin films converting moisture gradients or near-IR irradiation into motion.^32,33^ Nevertheless, these composite structures operate due to multistep processes or involve additional materials serving only as a mechanical scaffold. Accordingly, they are relatively slow with response times in the range of seconds to minutes.^29,30,32,33^

Here, we demonstrate for the first time fast light-to-motion conversion in ultrathin, pristine PDA membranes fabricaded by electropolymerization. We show that bare PDA membranes exhibit submillisecond contractions triggered by light and spontaneous expansion in dark conditions within milliseconds. These features are essential for building bottom-up, soft matter, ultrafast, nano-, and microphotoactuators. We have studied this phenomenon by investigating the effect of light, temperature, and moisture level with optical microscopy, reflectivity, Brillouin light scattering, and sum-frequency-generation spectroscopy. Our findings reveal that the contraction/expansion results from the desorption/adsorption of water molecules from/to the membranes.

## Results and Discussion

### Polydopamine Membranes

**Figure 1:**
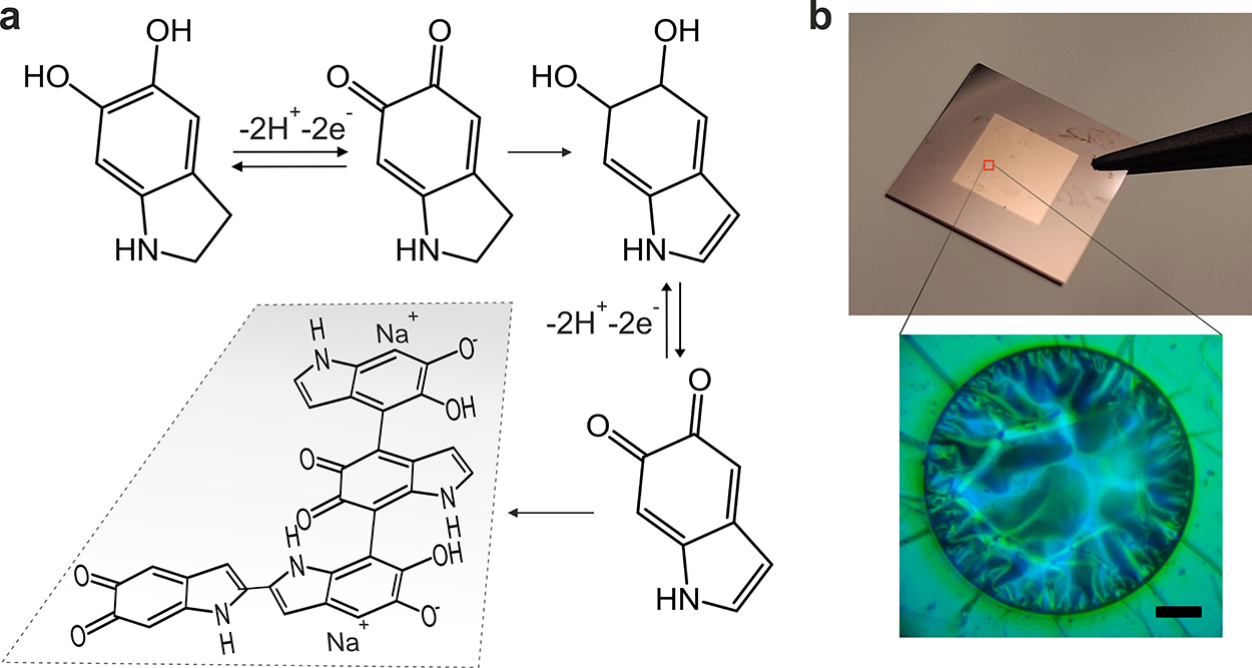
(a) The synthesis procedure of PDA with hydroxyl indole-like structures.^34^ (b) Optical images of the Si_3_N_4_ membrane grid covered with the PDA film (upper panel) and a free-standing PDA membrane (lower panel). The scale bar is 10 μm.

PDA films with homogeneous surface roughness (about 2.8 nm), thickness of about *t* = 15 nm, and Young modulus of about *E* = 12 GPa were prepared by electropolymerization.^34^ The PDA films were polymerized on a gold electrode surface using cyclic voltammetry, and the potential ranged between ±0.5 V at a scan rate of 2 mV/s. This relatively slow potential sweep has been set to promote the formation of the hydroxyl indole-like lammelar structure.^35^ The chemical reactions leading to PDA formation are shown in Figure 1a. After incubation in carbonate buffer to increase the cross-linking density, the film was desorbed from the gold surface through an electrochemical removal cycle^34^ and mechanically stripped from the surface using a sacrificial layer of poly(vinyl alcohol) (PVA). The film was transferred and suspended over circular holes of 60 μm diameter in a 1 μm thick Si_3_N_4_ membrane. After the film transfer, the PVA layer was removed by dissolution in water. Such a method allows the fabrication of ultrathin films in a homogeneous and reproducible fashion. Although ultrathin PDA films have been already prepared by different approaches,^36,37^ the advantage of our method is the preparation of large area membranes (possible up to mm scale) with outstanding mechanical properties and the tunability of the film properties by the synthesis parameters, such as scan rate (V/s) and ending potential. More details about sample preparation and characterization can be found in the Supporting Information 1 (SI 1) and elsewhere.^34^Figure 1b shows optical images of the Si_3_N_4_ membrane grid covered with PDA film and the morphology of the PDA membranes.

### Photoactuation

**Figure 2:**
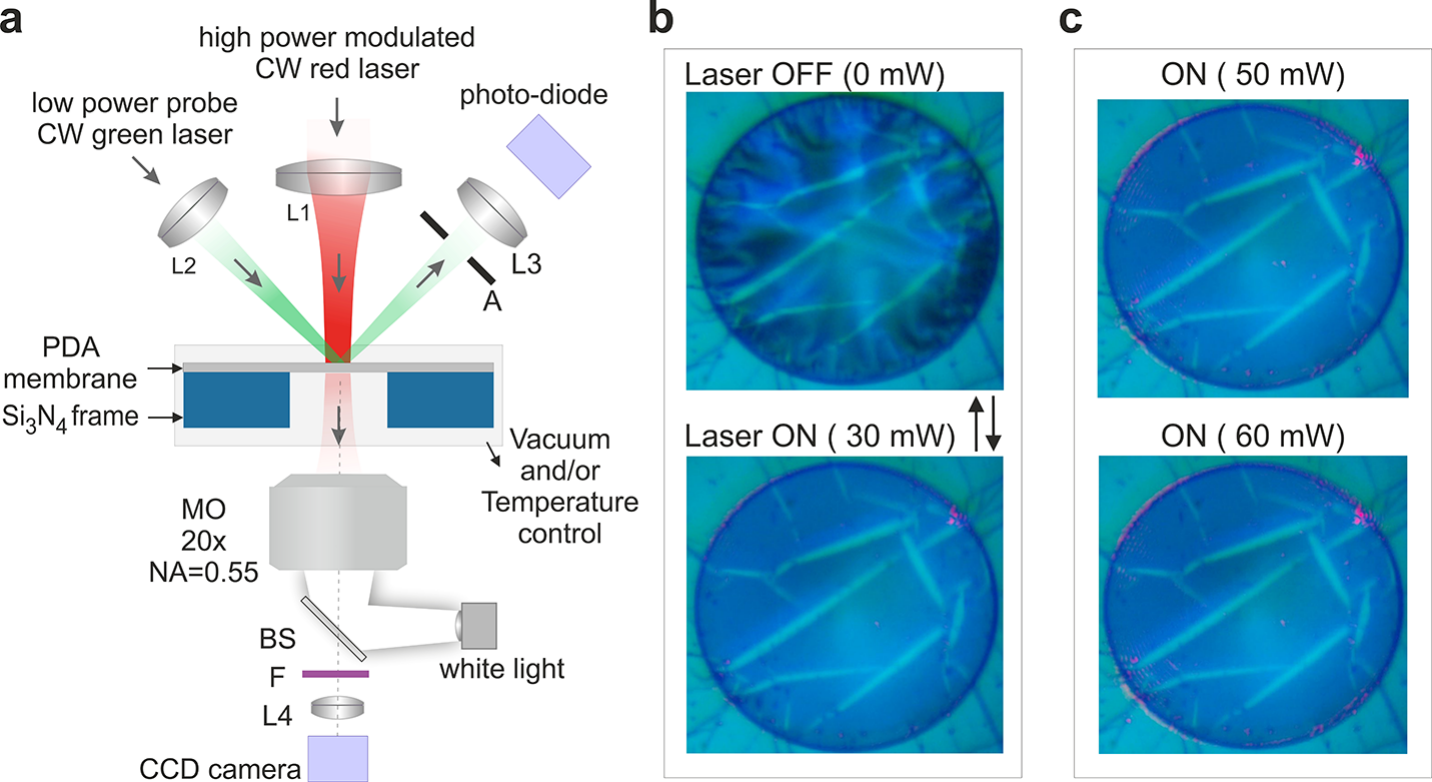
(a) Scheme of the experimental setup for the observation of light, temperature, or pressure-induced membrane actuation. The sample is mounted in a temperature- and pressure-controlled microscope stage and becomes illuminated with a red (660 nm) laser light that triggers the photoactuation. The laser light spot size approximately matches the membrane area. The change of the membrane morphology is visualized by a CCD camera. Reflectivity measurements using a low power green (532 nm) laser light are used to study fast morphological changes of the membrane. Symbols: L1, L2, L3, L4, lenses; F, optical filter; BS, beamsplitter; A, aperture; MO, microscope objective; CW, continuous wave. (b) Optical images of a PDA membrane subjected to light-induced contraction at ambient conditions. The power of the incident red laser light is 30 mW. The red laser light in the ON state is not visible due to the optical filter F. (c) The photoactuated state (laser ON) of the PDA membrane at 50 and 60 mW laser power (upper and lower panels, respectively).

The photoactuation of the PDA membranes has been triggered and observed with the experimental apparatus shown in Figure 2a, which is based on two continuous-wave (CW) lasers and a white light source. The deformation of the membrane was periodically stimulated with 660 nm CW laser light with 100 mW maximum power. The spot diameter of the stimulating red laser light matched the membrane size. At this wavelength, the measured light absorption of the PDA membrane (15 nm thick) was 4.4%. Taking into account the membrane thickness, the measured absorption of the PDA membranes is in accordance with the optical^38^ and photothermal^25−27^ properties of PDA (SI 2).

The light power of the red laser was modulated using a square waveform signal (on–off keying) with varied frequency (1–100 Hz) and amplitude (10–100 mW). The deformation of the membrane was directly observed with optical microscopy using low intensity white light illumination. Simultaneously, the mechanical response of the membrane was probed with a low-power (<1 mW) green laser light (532 nm) reflected from the membrane and detected by a photodiode.

Figure 2b shows the response of the PDA membrane to the illumination with the red light as observed with optical microscopy. In the absence of light (OFF), the surface of the membrane was buckled with dense wrinkles (upper panel in Figure 2b). Irradiation (ON) at the center of the membrane [lower panel in Figure 2b and Supplementary Movie 1 (SM 1) with 30 mW power caused an immediate flattening of the surface. The same phenomenon was observed for different light powers (SM 2). The buckling and wrinkles rapidly re-emerged once the red laser light was switched off and the membrane returned to the initial state. Figure 2c shows the flat (laser ON) state of the PDA membrane at 50 mW (upper panel) and 60 mW (lower panel). From these images, it becomes evident that the surface morphology of the irradiated PDA remains unchanged. By further increasing the red laser power the light-driven motion stayed reversible up to 90 mW (SI 3).

### Dynamics of Photoactuation

**Figure 3:**
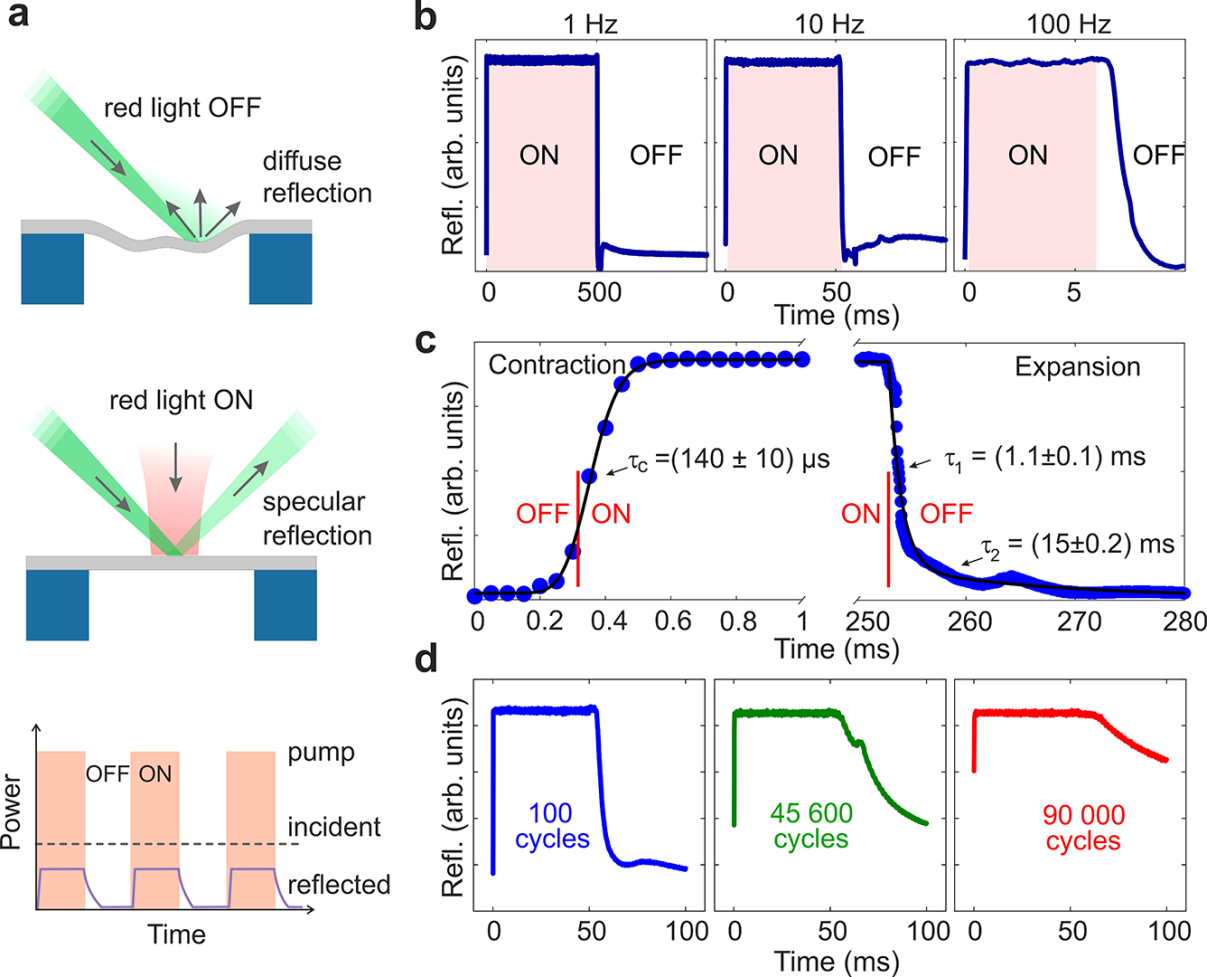
(a) Principle for measuring the dynamics of wrinkles with time-resolved reflectivity of the membranes. Red laser light ON triggers the flattening of the membrane and specular reflection of the green light (upper panel), whereas the red laser OFF state corresponds to diffuse reflection of the green light (middle panel). In the lower panel, “incident” and “reflected” intensities refer to the green light used to probe the membrane motions, and the red-shaded areas mark the temporal intervals when the red pump laser is on. (b) Periodic contraction and flattening of an irradiated PDA membrane. The membrane becomes flat for 0.5 s, 50 ms, and 6 ms with a repetition rate of 1, 10, and 100 Hz, respectively. (c) Left panel: time-trace of laser-driven contraction and flattening during the first 1 ms (blue points) and fitting with an exponential rise (black line). Right panel: Relaxation via swelling and wrinkling/buckling from 250 to 280 ms (blue points) and fitting with a biexponential decay (black line). The red vertical lines mark the start and end of the laser irradiation, respectively. (d) Fatigue testing of PDA membranes subjected to laser-induced contraction. The sample was periodically irradiated for 50 ms every 100 ms with 10 mW incident power of laser light.

To elucidate PDA contraction and expansion dynamics, we utilized the reflectivity experiment depicted in Figure 2a. Figure 3a illustrates how the reflectivity measurements can capture fast morphological changes of the PDA membranes and reveal the dynamics of photoactuation. Initially (red laser OFF, upper panel of Figure 3a), the membranes were buckled with wrinkles resulting in diffuse reflection of the green probing light and suppressed photodiode signal. The membrane contracts and flattens when exposed to high-power laser irradiation (red laser ON, central panel of Figure 3a). This behavior results in specular reflection of the green laser light captured by the photodiode. Even though the membrane presented some folded parts (Figure S5), the obtained signal was not influenced and a clear and reproducible response signal was obtained. The lower panel of Figure 3a illustrates a schematic of the duty cycle of the pump laser, the sample response, and the time-evolving intensity of the reflected light from the membrane. Figure 3b displays the membrane reflectivity as a function of time in response to three exemplary modulated (on–off keying) frequencies of the red laser light (SM 3).

The contraction and flattening of the membrane happened in submillisecond time scales after the laser is switched-on (Figure 3c, left panel). The reflectivity data have been represented with exponential decay functions to extract the response time of photoactuation and relaxation (SI 4). On the basis of this procedure, the time-constant for contraction and flattening is found to be τ_c_ = (140 ± 10) μs, which approaches the instrumental time-resolution. Notably, the observed response to the light stimulus is substantially faster than in the prior studies on, for example, NTE polymers,^39^ photochemical actuators,^10^ PDA-coated liquid crystal elastomers,^31^ and PEDOT-Tos bilayer structures^40^ (100–1000 s, 1–10 s, 0.1 s, and 3 ms, respectively) and comparably fast with VO_2_-based actuators (milliseconds to 160 μs).^9,41^

The membrane remained flat as long as the driving laser light was on. Once the red light was switched off, the sample relaxed back to the buckled state with slower relaxation lasting about 20 ms, as shown in the right panel of Figure 3c. The relaxation dynamics can be represented with a biexponential function with time-constants τ_1_ = (1.1 ± 0.1) ms and τ_2_ = (15 ± 2) ms. The biexponential dynamics are compatible with a two-stage mechanism where a fast water adsorption is followed by slower structural relaxation. Such two-stage dynamics has been observed during polymer-swelling, albeit the typically reported time scales are slower. For instance, the water adsorption and structural relaxation of zwitterionic polymer films (61 nm thick when dry) lasted few seconds and 20 min, respectively.^42^ The aqueous swelling of PDA coatings (79–85 nm thick) is completed in 120 min or more depending on the cross-linking density.^28^

One of the key parameters of actuating materials is fatigue due to cyclic loading. Therefore, we subjected the membranes to loading over a certain number of light-induced cycles with 10 Hz frequency. Figure 3d shows that periodic irradiation with 10 mW of the incident red laser light could keep the PDA membrane in motion even after 45 600 and 90 000 cycles. The time-constant for the contraction remains around 0.1 ms throughout cycling. Nevertheless, the relaxation of the membranes revealed some fatigue after prolonged actuation. Possibly, a long irradiation cycle caused additional cross-linking of PDA^28^ and reduced water adsorption.

### Thermal Stimulus

**Figure 4:**
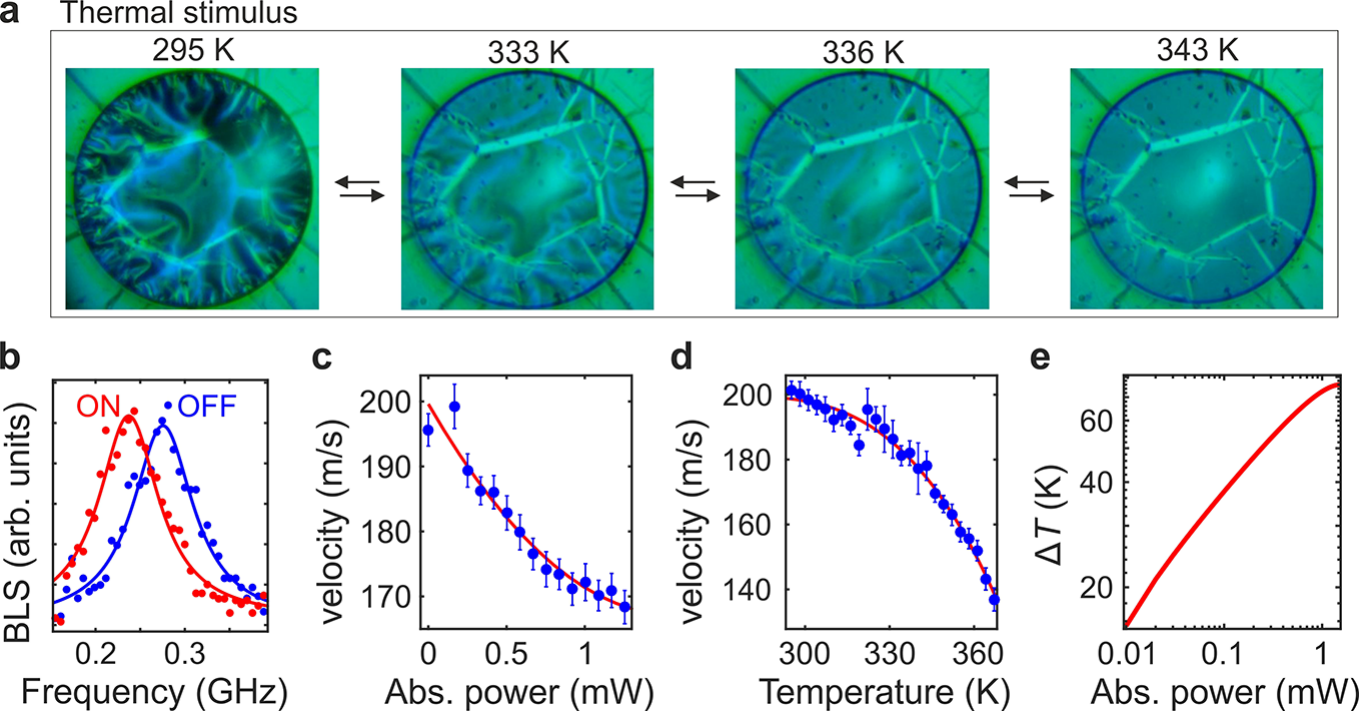
(a) Optical images of PDA membrane at exemplary temperatures during the heating–cooling cycle. (b) The BLS spectra of PDA membranes with the 660 nm laser light either off or on (30 mW). (c,d) The dependence of the A0 wave frequency on the absorbed laser power and the temperature of PDA, respectively. Experimental data and polynomial fittings are shown with blue points and red lines, respectively. (e) The calculated laser-induced temperature rise (Δ*T*) as a function of the absorbed laser power.

To elucidate whether the contraction of the membrane was due to photothermal or photochemical processes, we subjected PDA membranes to uniform heating. The temperature was gradually increased from 295 to 367 K (10 K/min) at ambient pressure. The optical images of the membranes captured at selected temperatures are displayed in Figure 4a (SM 4). Here, we can notice that the wrinkles start vanishing at about 333 K and the membrane is fully flattened at 343 K. This process was fully reversible, that is, cooling to room temperature returned the membrane surface to the initial buckled state. Hence, we concluded that the origin of the contraction was not photochemical as the same effect was achieved with a temperature rise of about 40 K above room temperature. Conclusively, the red laser light absorbed by the membrane serves as a local heat source.

To determine the laser-induced temperature rise in the membrane, we employed Brillouin light scattering (BLS). BLS is an inelastic light scattering technique that can be used to study hypersonic acoustic waves/phonons, elastic properties,^43^ and photoinduced phenomena^44^ of semiconducting nanomembranes. The BLS measurements were carried out with a CW laser with wavelength λ = 532 nm, low power (0.5 mW), in backscattering geometry and with an angle of incidence θ = 22° corresponding to the acoustic wavenumber *q* = 4π sin θ/λ = 8.85 μm^–1^. Figure 4b shows the BLS spectra obtained for the PDA membrane at room temperature with the red laser off and during red laser light irradiation. The illumination of the membrane with the intense red light causes a red-shift of the peak corresponding to the flexural (A0) acoustic wave.^34,43^ The spectral position of the A0 wave was measured as a function of the absorbed power of the red laser light. The measured frequency *f* and the phonon wavenumber *q* are then used to extract the phase velocity *v* = 2*πf*/*q* of the A0 wave. The results plotted in Figure 4c reveal systematic reduction of *v* while increasing the absorbed light power. This effect can be attributed to elastic softening of the membrane and changes of thickness due to heating.

Figure 4d displays the temperature dependence of *v* measured under uniform heating and the red laser light switched off. Noticeably, the global temperature rise results again in reduced velocity of the A0 wave, similar to the laser irradiation. These two experiments can be used to calculate the local temperature rise in the irradiated membranes (Figure 4e). For the incident laser power of 30 mW (as in Figure 2b) the local temperature rise is about 75 K. Conclusively, the laser-induced heating is sufficiently high (>40 K, Figure 4a) to eliminate surface wrinkles. Thus, the laser-induced PDA contraction can be solely explained by the photothermal effect.

Furthermore, the results of Figure 4c–e are compatible with reduction of the PDA thickness upon laser irradiation or temperature increase. The velocity of the A0 wave is proportional to both the Young modulus *E* and the thickness of the membrane^43^*t* as *v* ∝ *E*^1/2^*t*^3/2^. The results in Figure 4c show that with a incident laser power of 30 mW (1.3 mW absorbed) *v* decreased by 18%. This effect can result either from a 33% decrease of *E* or a 12% decrease of *t*. However, prior studies showed that the Young modulus of PDA significantly increases with heating.^45^ Thus, the observed reduction of *v* in Figure 4c,d can be explained by membrane contraction, which corroborates with the results of optical microscopy.

### Moisture Level Stimulus

**Figure 5:**
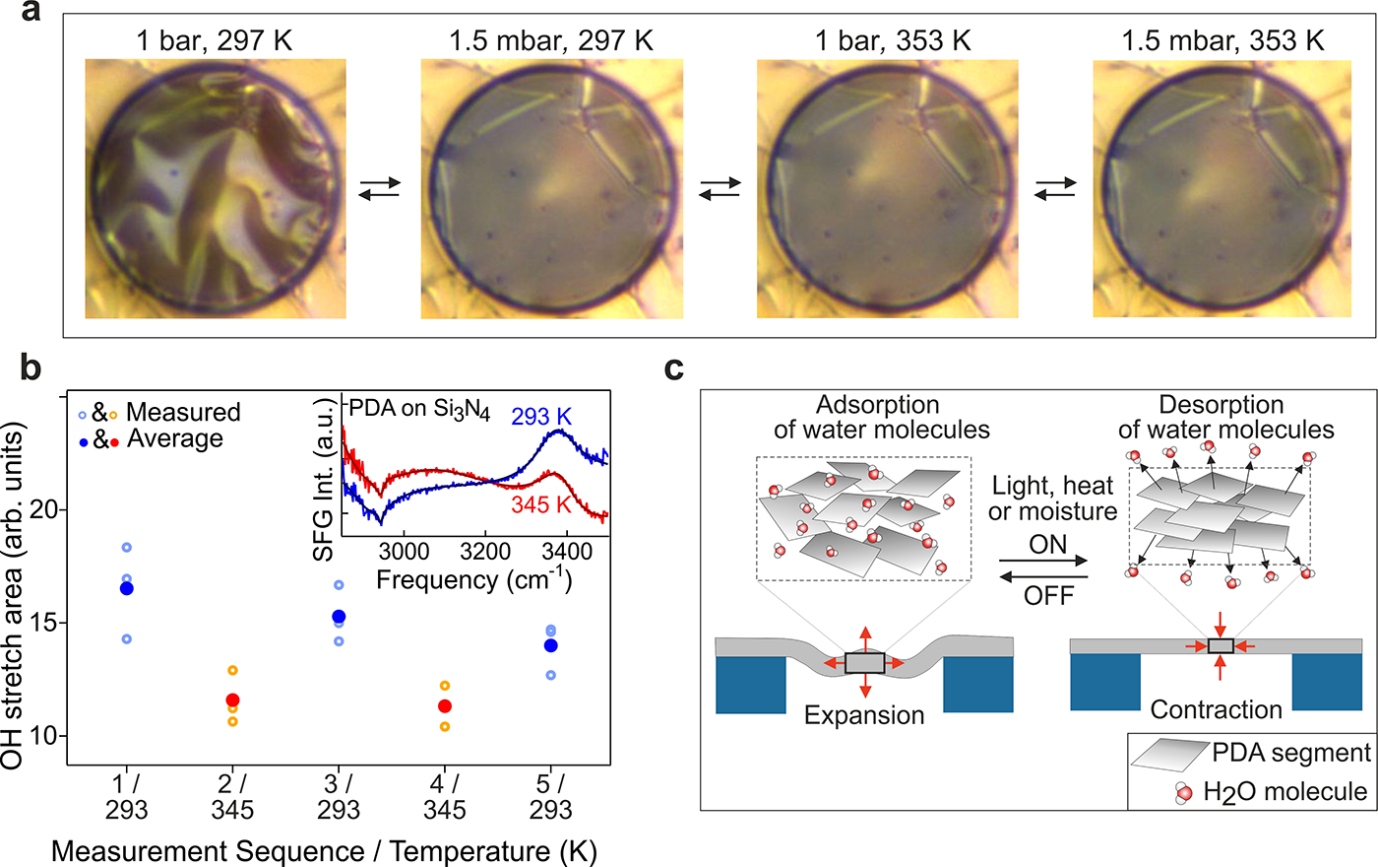
Optical images of a PDA membrane at (from left to right) room temperature and ambient atmosphere, room temperature and 1.5 mbar pressure, and temperature of 353 K and pressure of 1 bar and 1.5 mbar, respectively. (b) Summary of the individual and average fitted OH band area during two temperature cycles. The insert displays SFG spectra of PDA deposited on Si_3_N_4_ at 293 K (blue) and 345 K (red) and their corresponding fit curves. Both spectra show a CH stretch and OH stretch band, with the area of the OH band decreasing upon heating. (c) A scheme of PDA membrane contraction/expansion due to light-, heat- or moisture-induced desorption/adsorption of water molecules. The insets show the proposed microscopic lamellar-like structure of PDA^35^ that can adsorb (left) or desorb (right) water molecules from the atmosphere.

The photothermal effect that drives contraction of PDA can be related with negative thermal expansion (NTE)^39^ or moisture desorption/adsorption.^46^ To distinguish between these two possibilities, we investigated the behavior of the membrane under low air pressure, which reduces moisture (Figure 5a). Figure 5a (left panels) displays optical images captured at ambient pressure of 1 bar and 1.5 mbar at 295 K, corresponding to a relative humidity of about 60% and 0%, respectively. The decrease of the air pressure resulted in membrane flattening already at room temperature. The full reversibility of the process indicates that desorption/adsorption of water causes PDA contraction/expansion. Notably, heating of the (dehydrated) membrane in vacuum from 295 to 353 K (rightmost panel of Figure 5a) did not lead to any visible wrinkles. Thus, the intrinsic thermal expansion of PDA was negligible in our study.

In order to investigate the molecular changes that could occur in the PDA membrane upon thermal switching, sum-frequency-generation (SFG) vibrational spectra of PDA deposited on Si_3_N_4_ were measured. This technique allows the investigation of the polymer in a highly surface sensitive and chemically specific manner.^47,48^ SFG spectra (inset in Figure 5b) taken at 293 and 345 K show bands at 2950 and 3400 cm^–1^ attributed to polymer CH stretch modes and to OH stretch modes of the polymer and/or of water, respectively. The area of the OH-stretch band (Figure 5b; SI 5) decreases upon heating and recovers upon subsequent cooling. Some dampening of the effect due to polymer aging during the prolonged exposure to laser radiation is observed. While spectral contributions of the OH groups of the polymer and the water adsorbed in the film cannot be decomposed, the observed changes are consistent with a reversible temperature-induced change to the structure of the polymer and with the hypothesis of water removal.

Figure 5c illustrates the proposed microscopic mechanism manifested as the macroscopic contraction of PDA in response to external stimuli. The relatively slow potential sweep that was used during the electro-polymerization of PDA favors the formation of a hydroxyl indole-like lamellar structure.^35^ Thus, in the present work, ultrathin PDA is expected to contain short semicrystalline molecular segments of 5–6 hydroxyindole (DHI) units held together by weaker intermolecular bonds and water molecules trapped in the free volume that remains after cross-linking. In this work, the structural characterization with HR-TEM and FTIR is not feasible due to the small thickness of the membranes. However, quasi-crystalline graphenelike molecular segments of PDA have been observed in thicker specimens.^49,50^ We propose that the adsorption (desorption) of water can strengthen (weaken) the intermolecular interactions in PDA, allowing in this way the rearrangement of the DHI crystalline unit and leading to the observed light-driven contraction. In ambient conditions, the membrane adsorbs moisture leading to increased distances between the PDA segments and expansion. Desorption of water from PDA, driven by light, temperature changes, or vacuum, causes collapsing of PDA segments and macroscopic contraction of the membrane. Interestingly, the temperature at which the membranes become flat (i.e., 370 K in Figure 2b and 333–343 K in Figure 4a) match the temperature for desorption of surface-bound water from PDA (340–350 K).^28,51^

## Conclusions

Herein, we demonstrated for the first time fast photoactuation of bare polydopamine membranes fabricated with state-of-the-art electropolymerization and PVA-assisted transfer onto holey substrates. With this method, PDA exhibits photoactuation without requiring additional mechanical or photothermal components as in prior studies.^29−33,40^ Visible light drives ultrafast contraction (<140 μs) of the membranes, while switching off light leads to spontaneous expansion in milliseconds time scale. The ultrafast response time is attributed to the small inertia of the membrane, fast water desorption due to the small thickness, and rapid heat transfer to the surroundings (see also SI 6). The stimulated contraction mimics natural muscle fibers’ behavior as opposed to the most frequently used photoactuators based on thermal expansion and bending.^41,52^ Contraction/expansion of the membranes can also be driven by heat and moisture. The observed phenomenon is attributed to the desorption/absorption of water molecules by PDA to/from the atmosphere. The proposed explanation is in line with the effect of molecular penetrants known for swelling of polymers^42^ including PDA.^28^

Bare PDA membranes made by electropolymerization can be used as multistimuli building blocks for soft micro- and nanodevices.^41,52^ PDA offers broad-spectrum photothermal features, relatively high Young modulus,^34^ and strong adhesion on multifarious surfaces,^17,53^ making it useful for nanoscale remote actuation, artificial muscles, moisture or light sensing, energy harvesting, and adaptative optics or metamaterials (photonic and phononic). Moreover, the actuation of wrinkles on membranes can be used in pressure- and strain-sensitive, moisture-erasable, vibrotactile displays, and data-recording devices.^40,54^ Finally, PDA can be patterned and decorated with metals using lithographic techniques,^53^ which can be useful for the mass production of nanorobots or other nanodevices.^55^ We envisage that this work will stimulate further fundamental studies on actuation by nonmonochromatic light, optimization of the preparation conditions, and the detailed microscopic picture of the water dynamics in PDA.

## Supporting Information Available

Additional information on the (i) preparation and characterization of the PDA membranes (cyclic voltammetry, grazing incidence angle FTIR, and atomic force microscopy), (ii) optical properties of the membranes, (iii) damage threshold for laser irradiation, (iv) processing of the time-resolved reflectivity data, and (v) sum-frequency-generation spectroscopy measurements and their analysis, (vi) reflectivity dynamics of larger membranes (PDF)
Movie of optical microscopy of 30 mW irradiation (MP4)
Movie of irradiation with various laser powers (MP4)
Movie of irradiation with various on–off keying frequencies (MP4)
Movie of PDA membrane subjected to uniform heating (MP4)
