# Supplementary material for: Fast Light-Driven Motion of Polydopamine Nanomembranes
Source: Nano Lett. 2021 Dec 14;22(2):578–85. doi: 10.1021/acs.nanolett.1c03165 (PMC8796235; doi:10.1021/acs.nanolett.1c03165)
Supplement: Supplementary file 1 — nl1c03165_si_001.pdf [file nl1c03165_si_001.pdf]

Supplementary Information:
Fast light-driven motion of polydopamine nanomembranes

Thomas Vasileiadis[1,2], Tommaso Marchesi D'Alvise[2], Clara-Magdalena Saak[2,3], Mikolaj Pochylski[1], Sean Harvey[2], Christopher V. Synatschke[2], Jacek Gapinski[1], George Fytas[2], Ellen H.G. Backus[2,3], Tanja Weil[2,*], and Bartlomiej Graczykowski[1,2,*]

1. Faculty of Physics, Adam Mickiewicz University, Uniwersytetu Poznanskiego 2, 61-614 Poznan, Poland
2. Max Planck Institute for Polymer Research, Ackermannweg 10, 55128 Mainz, Germany.
3. Department of Physical Chemistry, University of Vienna, Währinger Strasse 42, 1090 Vienna

* weil@mpip-mainz.mpg.de
* bartlomiej.graczykowski@amu.edu.pl

1. Preparation and Characterization of PDA membranes.

Polydopamine (PDA) films were polymerized on a gold electrode surface using cyclic voltammetry (see **Fig. S1** and **section 1.1**). Grazing incidence angle FTIR was applied to characterize the prepared films before and after transfer from the gold electrode surface (**section 1.2**). Before and after transfer, both films revealed similar thickness of 16 to 14 nm, respectively, as determined by AFM (see **section 1.3**).

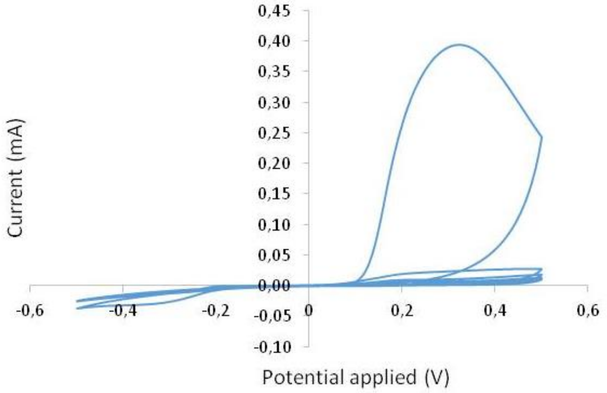

Figure S1: Cyclic voltammogram for polydopamine film formation. Cyclic voltammogram of PDA film in phosphate buffer pH 7 where the potential was swept between +0.5 and –0.5 V for 5 cycles starting and ending at 0 V vs Ag/AgCl reference electrode with a scan rate of 2 mV/s. The first oxidation peak for the reaction from catechol to quinone form of dopamine molecule can be distinguished at 0.3 V. Other oxidation and reduction peak are barely present due to the slow scan rate.

1.1 Electropolymerization of PDA membranes.

Materials

Gold-coated (1000 Å) microscope slide (Sigma-Aldrich), were cut using a diamond tip. Phosphate buffer pH 7, 100 mM was prepared using sodium phosphate dibasic anhydrous (99%) and sodium phosphate monobasic (99%) (Sigma-Aldrich) in Milli-Q water. Carbonate buffer pH 10, 100 mM was prepared using sodium bicarbonate (>99.7%) and sodium carbonate (>99.8%) from Sigma-Aldrich, in Milli-Q water. Polyvinyl alcohol 80% hydrolyzed 9–10 kDa Mw (Sigma-Aldrich) was used to prepare the 10 wt% solution in Milli-Q water. Custom-made silicon nitride substrates were purchased from Silson and Norcada.

Method

Electropolymerization was performed using a Metrohm Autolab N series potentiostat (AUTOLAB PGSTAT 204) and a three-electrode configuration. A gold-covered glass microscope slide was used as the working electrode, a Ag/AgCl with 3M KCl as the reference electrode, and a gold wire as the counter electrode. All reactions were performed in a 30 mL electrochemical cell (Metrohm) filled with 25 mL of solution in air atmosphere and at room temperature. The working electrode was pre-treated for 10 minutes with Ar plasma cleaning at a pressure of 6 mbar. The gold substrate was immersed in a 1 mg/mL solution of dopamine hydrochloride dissolved in 100 mM phosphate buffer at pH 7.0 previously bubbled with Nitrogen to remove oxygen from the solution and prevent dopamine self-polymerization. The film was then transferred either to silicon support for AFM measurements or to Si_3Ni_4 substrates for actuation and SFG experiments through a previously established transfer method[1].
Briefly, PDA films were first incubating in carbonate buffer at pH 10 for 30 min before applying a removal cycle by sweeping the potential between -0.8 and +1.2 V for 3 cycles with a scan rate of 20 mV/s to desorb it from the gold surface. The film was then covered with a polyvinyl alcohol (PVA) 80% hydrolyzed solution, 10% mass in water, and once dried for ca 25 min in the oven at 40 ºC, it was stripped off the surface and transferred onto the respective substrates. The PVA layer was removed by washing with water. Before the SFG experiments, the PDA film already transferred on Si_3N_4 was incubated in the oven at 70 ºC for 30 min.

1.2 Grazing incidence angle FTIR.

The infrared spectrum of the film on gold obtained by grazing-angle reflectance FTIR (Vertex 70, Bruker) after purging the sample with dry air for 15 minutes and recording 4 spectra at 3000 scans with an interval of 1 minute between each one.

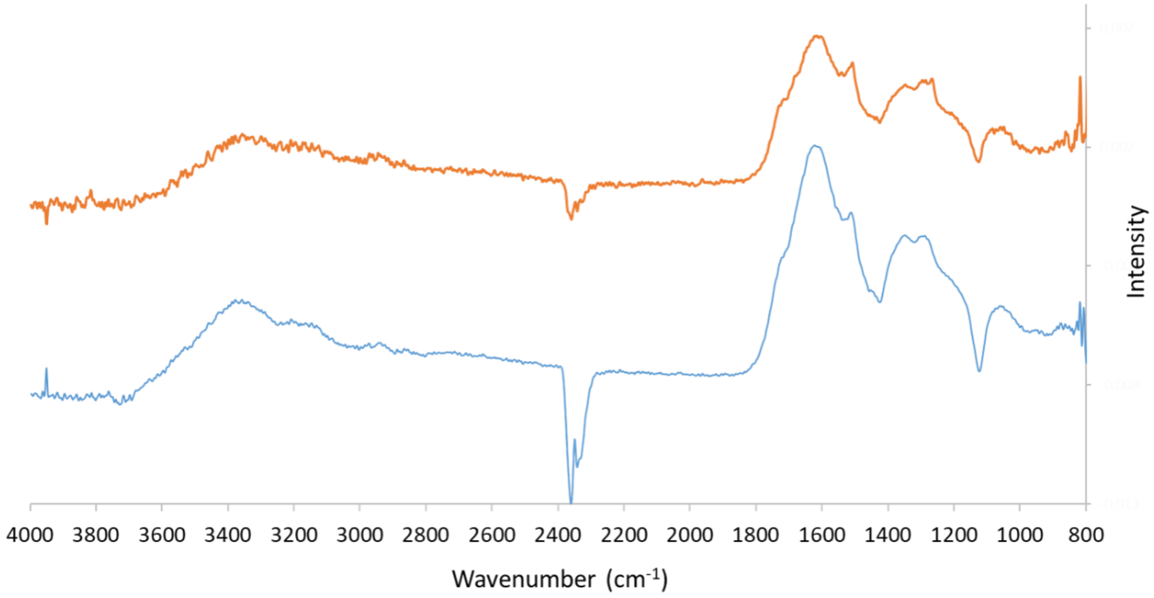

Figure S2: Grazing incidence angle FTIR. Spectra of polydopamine film 5 cycles before (orange) and after (blue) the carbonate buffer incubation and the removal procedure was performed.

1.3 Atomic force microscopy.

Atomic force microscopy was used to characterize the film, measuring the morphology, thickness of the polymer films on gold by scratching it with a plastic tip after it was freshly prepared and after the transfer on Si support. The profile was then recorded by AFM (Park NX20 and Bruker Dimension ICON) with a cantilever 70 KHz resonance frequency and an elastic constant of 2 N/m in tapping mode. The film freshly prepared on gold shows a mean surface roughness of 2.84 nm while the one transferred on silicon substrate of 2.7 nm. The profile and the roughness were measured by analyzing the images using the Gwyddion software on a region of ca 1.5 x 3.0 µm.

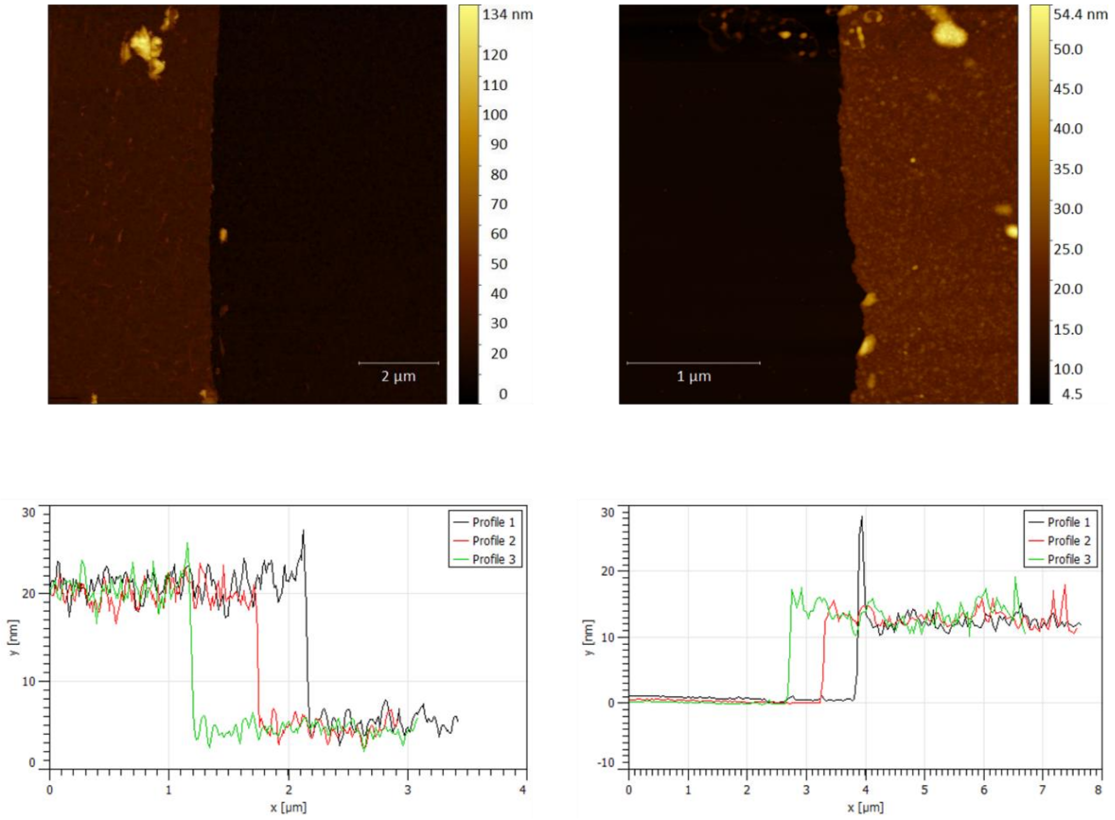

Figure S3: AFM images and height profiles. Atomic force micrograph of the film on gold (left) and transferred on Si substrate (right) and the corresponding profiles (down).

1.4 Wrinkle formation.

The nanoscopic wrinkles that are present on the film are formed during the transfer procedure. Such wrinkles arise already when the removal procedure is applied on gold even before removing the film from the surface, and are probably due to the partial desorption of the film from the substrate (**Fig. S4**). Wrinkles are mostly noticeable when the sample is transferred on a continuous substrate and not as much on free-standing membranes (**Fig. S5**).

The film is also showing stripes, which are folds that can occur once the membrane is transferred to a substrate, due to the manual transfer procedure, such folds are twice thicker than the membrane (see AFM Figs. **S4** and **S5**) confirming that the film is folding on itself. Finally, macroscopically visible wrinkles are induced during temperature treatment to trigger the phenomena, which are then disappearing upon photoactuation. Interestingly the higher magnification AFM of the film surface shows a porous structure (**Figure** S5D).

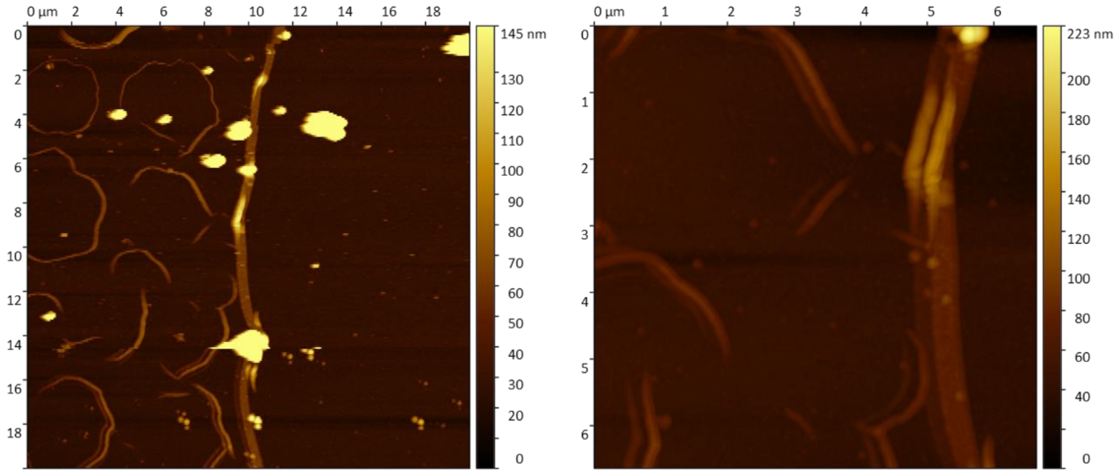

Figure S4: Wrinkle formation and visualization with AFM. PDA film on gold after removal procedure was applied.; wrinkles with circular pattern are appearing due to the desorption from the substrate.

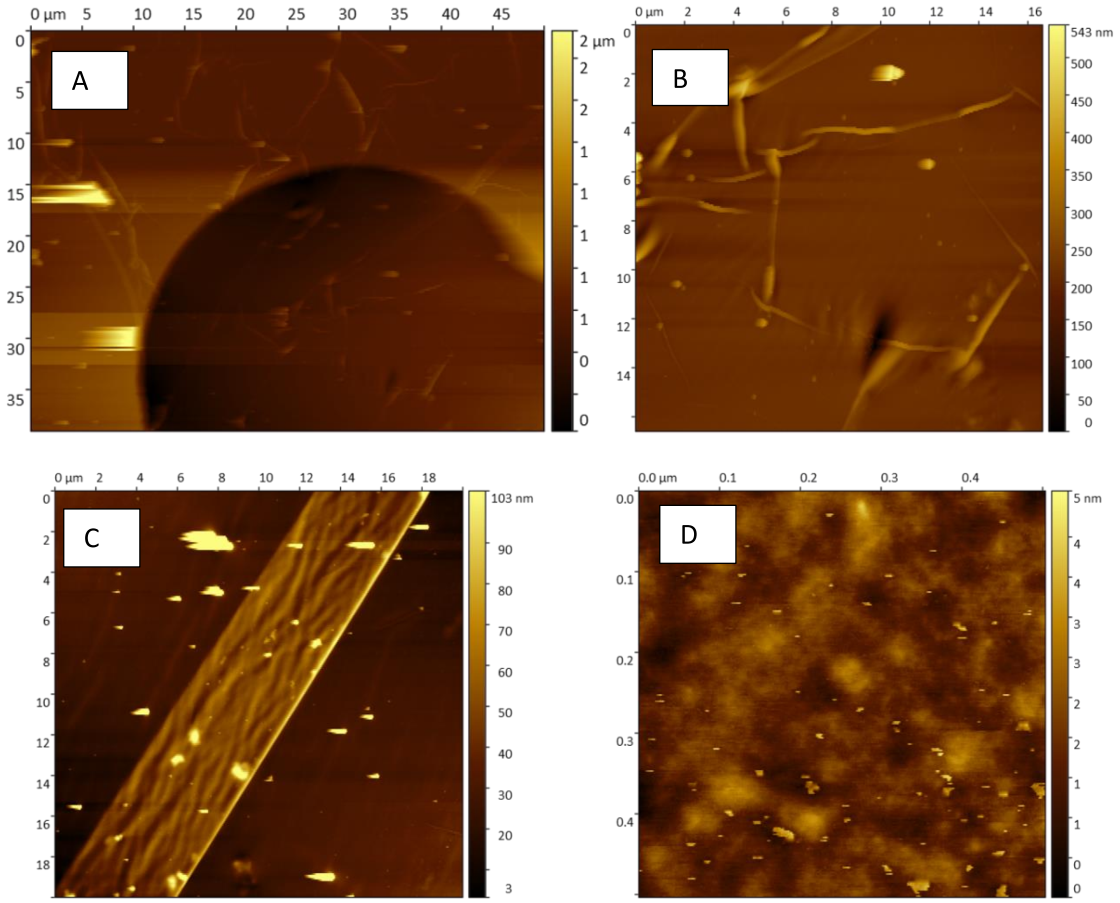

Figure S5: AFM microscopy of free-standing membranes. A. Micrograph of the free-standing membrane over a hole on the perforated Si_3N_4 film; **B**. Micrograph of the free-standing part where small wrinkles and folds are present;. **C**. micrograph of a fold present at the free standing film. **D**. Surface of the free standing film.

2. Optical absorption of PDA membranes

The absorption, thickness (τ) and penetration depth (δ) are connected with the formula $A_{(\%)} = 100 \cdot \left[1 - e^{-\tau/\delta}\right]$ (Beer–Lambert law) from which we derive $\delta = 0.33\ \mu m$ at 660 nm. These measurements are in good agreement with recent spectroscopic ellipsometry studies of various PDA thin films[2], which give $k = 0.1$ at λ=660 nm and $\delta = \lambda/4\pi k = 0.53\ \mu m$.

3. Laser power damage threshold

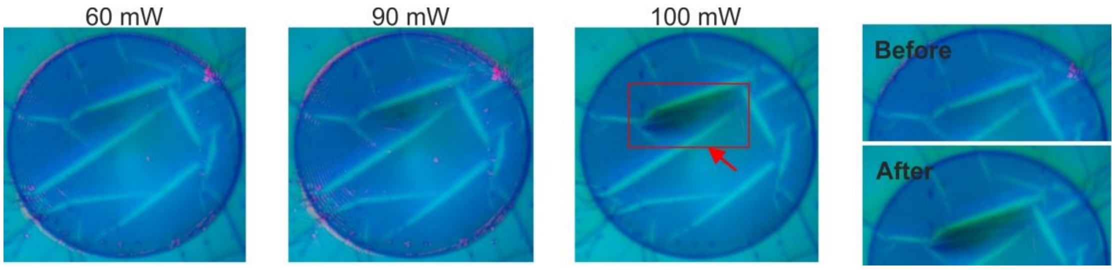

Figure S6: Optical microscopy images showing rack formation for laser powers larger than 90 mW.

4. Fitting of time-constants

To quantify how fast the response of the PDA membranes to laser light is, we have fitted the time-resolved reflectivity data with exponential decay functions of the form:

$$f(t) = \Theta(t - t_o) \cdot A \cdot \left(1 - \exp\left[-\frac{t - t_o}{\tau}\right]\right) \ ,$$

where t_o is the time that the laser turns on or off, $\Theta(t - t_o)$ is the Heaviside step function, A is the amplitude of the change, and τ is the time-constant. For fitting the experimental data, the $f(t)$ is convoluted with a Gaussian function, the width of which represents the time-resolution of the instrument, which is estimated to be 100 μs.

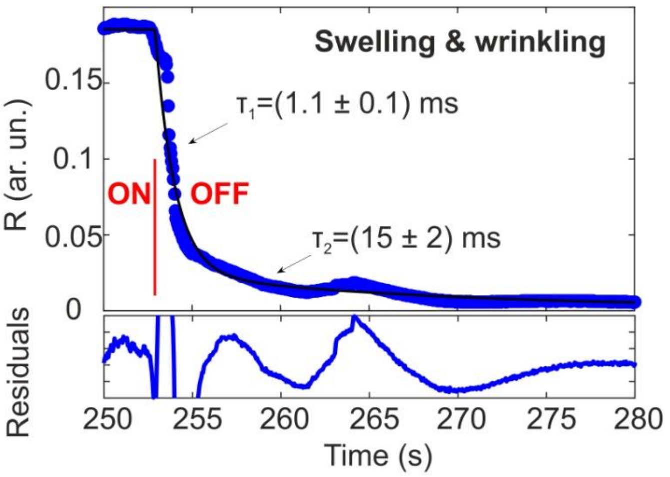

Figure S7: Bi-exponential fitting of relaxation (up) after the laser is switched off and residuals of the fit (down) showing additional oscillatory dynamics.

The time-constant for contraction and flattening is found to be τ_C=(140 ± 10) μs, which approaches the instrumental time-resolution. For this process the bioexponential fitting is adequately reproducing the data – see Fig. 3c of the main manuscript. The reverse processes of swelling and wrinkling can be represented with a bi-exponential function with time-constants τ_1=(1.1 ± 0.1) ms and τ_2=(15 ± 2) ms. For the relaxation (wrinkling and swelling) the signal contains oscillations in addition to the exponential dynamics (residuals in **Fig. S7**), which can originate from mechanical instabilities such as buckling and wrinkling.

5. Sum-frequency-Generation Spectroscopy

Methods

Laser light was produced by a Ti:Sapphire-based amplifier (Coherent Libra), yielding 40 fs pulses at 800 nm with a 1 kHz repetition rate and approximately 5 mJ in energy. Part of the laser output was passed through an OPA (TOPAS Prime) to generate infrared light (IR) at 3200 cm^{-1}. Another part passed through an etalon which narrowed the spectral width to ~20 cm^{-1}. The 800 nm and the IR pulses were subsequently overlapped on the sample in space and time to generate light at their sum frequency (SFG). The generated SFG signal was focused onto a spectrometer (Shamrock 303i) and detected on a CCD camera (Newton EMCCD Andor). The SFG data shown in this work was collected in SSP polarization. This experimental setup has previously been described by Schlegel et al.[3] As previously mentioned SFG involves the generation of light at the sum-frequency of two incident light beams. This nonlinear process is dependent on the second-order nonlinear susceptibility of the material in which the two beams are overlapped. Since the second-order nonlinear susceptibility becomes zero in centrosymmetric media, SFG is only allowed in the non-centrosymmetric parts of the investigated systems. In the case of the studied PDA film this means that the SFG

process is inherently surface sensitive. A more detailed summary of the SFG process can be found in the article of Lambert et al.[4]

In order to change the temperature of the polymer film, the sample is placed onto a copper block through which water is circulated at a defined temperature. The heating block is in turn placed on a 3D translational stage to bring the sample into alignment with the SFG pulse pair. The temperature of the circulating water is heated/cooled and circulated by a commercially available chiller. The set temperature of the chiller and the measured temperature on the surface of the copper block was 20/20 °C and 80/72°C, respectively. In total 5 set points, corresponding to two temperature cycles were measured (20 °C - 80 °C - 20 °C - 80 °C - 20 °C) to investigate the reversibility of any spectral changes due to temperature.

Data Treatment

Each SFG spectrum was background subtracted by a spectrum measured with the IR beam blocked and normalized to an SFG spectrum of quartz, which was obtained under identical conditions. Quartz spectra measured at the beginning and end of the temperature cycle confirmed the stability of the setup.

At each temperature point, 3 spectra were accumulated for 5 minutes each. These spectra were individually fitted. Since the Si_3N_4 substrate contributes a pronounced non-resonant background to the spectra, which is reproducibly affected by the sample temperature, a nonlinear function was used to describe the background signal. The observed OH and CH stretch bands were fitted using one Lorentzian function each. A similar fitting procedure has previously been used by, e.g., Schlegel et al.[2], with the only difference being the nonlinear frequency dependence of the amplitude used to fit the non-resonant background signal.

The area of the Lorentzian function corresponding to the OH stretch band was then extracted and averaged at each temperature set point during the cycle to yield the values summarised in Table 1. The OH stretch mode of the PDA film was found at 3371(4) cm^{-1} with a line width of 125(8) cm^{-1}. Correspondingly, the CH stretch mode was found at 2938(5) cm^{-1} with a width of approximately 32(17) cm^{-1}.

Table 1: Summary of fitted OH stretch area

**Set/Sample Temperature**	**Cycle Nr.**	**Spectrum Nr.**	**Fitted Area**	**Average area**
[°C]	[#]	[#]	[a.u.]	[a.u.]
20 / 20	1	20_1	18.34	16.53
		20_2	16.95	
		20_3	14.29	
80 / 72	2	80_1	12.91	11.59
		80_2	10.64	
		80_3	11.23	
20 / 20	3	20_4	16.68	15.29
		20_5	15.00	
		20_6	14.19	
80 / 72	4	80_5	12.23	11.32
		80_6	10.41	
20 / 20	5	20_7	14.71	14.00
		20_8	14.61	
		20_9	12.69	

6. Response of larger membranes

The reflectivity experiment shown in Figure 3 of the main manuscript have been repeated for a PDA membrane of 80 μm diameter. The larger PDA membranes show the same behaviour albeit the dynamics for initial contraction are slower, in the millisecond timescale – see **Figure S8**.

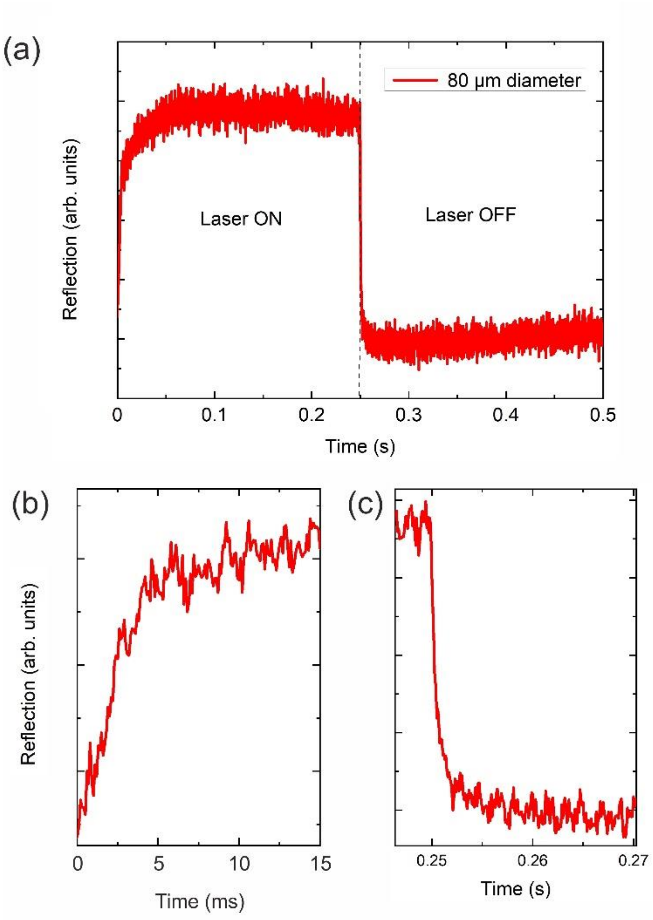

Figure S8: The light-driven motion of a wider (80 µm diameter) PDA membrane measured with time-resolved reflectivity (532 nm). (a) Periodic contraction and flattening of the irradiated PDA membrane with the driving laser (660 nm) on for 0.25 s and with a repetition rate of 2 Hz. (b) The initial contraction of the wider membrane is completed in less than 5 ms. (c) The relaxation (expansion) of the wider membrane takes place within approximately 10-20 ms.

References

1. Marchesi D'Alvise, T. *et al.* Ultrathin Polydopamine Films with Phospholipid Nanodiscs Containing a Glycophorin A Domain. *Adv. Funct. Mater.* **30**, 2000378 (2020).

2. Qie, R., Zajforoushan Moghaddam, S. & Thormann, E. Parameterization of the optical constants of polydopamine films for spectroscopic ellipsometry studies. *Phys. Chem. Chem.* Phys. **23**, 5516-5526 (2021).

3. Schlegel, S. J. *et al.* How water flips at charged titanium dioxide: an SFG-study on the water–TiO_2 interface. *Phys. Chem. Chem. Phys.* **21**, 8956–8964 (2019).

4. Lambert, A. G., Davies, P. B. & Neivandt, D. J. Implementing the Theory of Sum Frequency Generation Vibrational Spectroscopy: A Tutorial Review. *Appl. Spectrosc. Rev.* **40**, 103–145 (2005).
